# The Effects of Kinesio Tape on Acute Ankle Sprain: A Systematic Review

**DOI:** 10.3390/jcm14051440

**Published:** 2025-02-21

**Authors:** Guido Bocchino, Daniele Grassa, Antonio Bove, Matteo Salvini, Rami Kaplan, Emidio Di Gialleonardo, Fabrizio Forconi, Giulio Maccauro, Raffaele Vitiello

**Affiliations:** 1 Department of Orthopedics, Ageing and Rheumatological Sciences, Fondazione Policlinico Universitario A. Gemelli IRCSS, Largo A. Gemelli, 8, 00168 Rome, Italy; dr.danielegrassa@gmail.com (D.G.); antonio.bove1212@gmail.com (A.B.); mattesalvini@gmail.com (M.S.); emidiodiggia@gmail.com (E.D.G.); giulio.maccauro@policlinicogemelli.it (G.M.); raffaele.vitiello@policlinicogemelli.it (R.V.); 2 Department of Orthopedics and Geriatric Sciences, Università Cattolica Del Sacro Cuore, Largo Francesco Vito, 8, 00168 Rome, Italy; ramibkaplan@gmail.com; 3 Clinic Villa Stuart, 00135 Rome, Italy; fabrizio.forconi@gmail.com

**Keywords:** ankle sprain, kinesio taping, pain management, edema control, functional recovery

## Abstract

**Background:** An ankle sprain is a frequent musculoskeletal injury, often leading to chronic instability and an increased risk of post-traumatic osteoarthritis. Kinesio Tape, an elastic adhesive tape, is widely used in rehabilitation for its supposed benefits in reducing pain, controlling edema, and improving ankle function. However, its effectiveness in managing acute ankle sprains remains debated. **Methods:** This systematic review aims to evaluate the evidence on the impact of Kinesio Taping on pain reduction, edema control, and return to sport in patients with acute ankle sprains. A systematic review was conducted in line with the PRISMA guidelines. The literature from PubMed, MEDLINE, and the Cochrane Library was searched for studies published from 2004 to 2024. Seven studies met the inclusion criteria, involving 247 patients with acute ankle sprains. Data on patient demographics, follow-up duration, Kinesio Taping protocols, and clinical outcomes were extracted and analyzed. **Results:** The review revealed mixed findings. Some studies reported short-term pain relief and reduced need for analgesics in the Kinesio Taping group, especially when combined with manual therapy. However, no significant differences were found in the edema control compared to bracing or casting. Functional recovery, as assessed by scales like the Karlsson scoring scale, showed no clear advantage for Kinesio Taping over other treatments. **Conclusions:** Kinesio Taping provides limited benefits for ankle sprain management, particularly in terms of long-term functional recovery and edema reduction. While it may offer short-term pain relief, it should be considered as an adjunctive treatment rather than a primary intervention.

## 1. Introduction

The objective of this systematic review is to assess the effectiveness of Kinesio Taping in the management of acute ankle sprains by following the PRISMA (Preferred Reporting Items for Systematic Reviews and Meta-Analyses) guidelines. An ankle sprain is one of the most common injuries affecting the musculoskeletal system, contributing to a significant socioeconomic burden due to high medical costs for diagnosis and treatment, as well as a reduction in work productivity [1]. The cost of managing a lateral ankle sprain (LAS) per individual can vary greatly, ranging from 360 to 1300 euros. In the Netherlands, around 187.2 million euros are spent each year for the treatment of ankle sprains [2,3]. Key risk factors include female gender, younger age, and participation in indoor or court sports [4]. Recurrent ankle sprains often lead to instability, with the probability of re-injury ranging from 12% to 47% and may progress to chronic ankle instability—a common condition. There is also a notable association between initial ankle sprains, chronic ankle instability, and the eventual development of post-traumatic osteoarthritis [5].

Given the importance of addressing ankle sprains to prevent chronic issues, common treatments for acute ankle injuries include cryotherapy, compression, immobilization, functional rehabilitation (e.g., taping, bracing), exercise therapy, and manual mobilization. Preventive strategies often involve exercise, taping, and bracing to enhance ankle performance. Elastic tape is frequently used in professional sports as per consensus guidelines [6].

Kinesio Tape, a unique elastic adhesive tape, can stretch up to 140%, mimicking the elasticity of the skin. It recoils after application, lifting the skin microscopically, which promotes lymphatic drainage and reduces discomfort. This effect increases the interstitial space, reducing inflammation and pressure, while improving blood and lymphatic flow in the affected area [7]. This method alleviates pressure and irritation on neurosensory receptors, alleviating pain [8]. This tape is also latex-free and features an adhesive that is 100% heat-activated acrylic. The 100% cotton fibers allow for evaporation and fast drying, making it waterproof; this allows for a wear time of 3 to 5 days and makes the treatment more cost-effective. The tape is typically applied over and around muscles to prevent over-contraction [9]. Kinesio Taping is thus viewed as a rehabilitative technique that supports injured muscles and joints, aiding in the recovery of sprained ankle.

Numerous studies have examined the impact of Kinesio Taping on sports injuries [10], musculoskeletal injuries [11], muscle strength [12], spinal stiffness [13] and knee function [14]. This review aims to comprehensively evaluate the available evidence regarding Kinesio Taping’s impact on key clinical outcomes, including pain reduction, edema control, and return to sport, in patients with acute ankle sprains. By integrating findings from various studies, this review seeks to clarify the therapeutic role of Kinesio Taping and provide clinicians with evidence-based recommendations for optimizing the treatment of acute ankle sprains.

## 2. Materials and Methods

This systematic review was performed in accordance with the Preferred Reporting Items for Systematic Reviews and Meta-Analyses (PRISMA) guidelines, ensuring a thorough and systematic approach to data collection and analysis. Eligibility criteria included studies that evaluated the effects of Kinesio Taping in acute ankle sprains, reporting outcomes related to pain, edema, or functional recovery in adult patients with clinically diagnosed injuries. Searches were conducted in the PubMed, MEDLINE, and Cochrane Library databases up to April 2024, with only English language articles considered.

The study selection process involved two independent reviewers who screened studies for eligibility and resolved any conflicts through discussion. Data extraction focused on demographics, intervention protocols, and clinical outcomes. Pain reduction, edema control, and functional recovery were the primary outcomes analyzed, while secondary outcomes included return-to-sport rates and the need for analgesics.

The risk of bias in individual studies was assessed using the Cochrane risk of bias tool, with discrepancies resolved by consensus. Mean differences with confidence intervals were used to report outcomes. Due to heterogeneity in study designs and outcomes, a qualitative synthesis was conducted instead of a meta-analysis. A compliance statement was included to confirm adherence to the PRISMA 2020 guidelines.

### 2.1. Search Strategy

In this study, a systematic review of the literature indexed in the PubMed, MEDLINE, and Cochrane Library databases using the keywords “tape” or “taping” and “ankle sprain” was performed in April 2024.

To minimize the number of missed studies, no filters were applied to the search strategy. The literature was limited to publication from 2004. The only restriction applied was the language (only full-text articles in English). The title of the journal, the name of authors, or supporting institutions were not masked at any stage. No attempt to contact the authors to obtain individual patient data was made. The authors excluded cadaveric and animal studies.

### 2.2. Inclusion and Exclusion Criteria

The reviewers (G.B., D.G., A.B., E.D.G., and R.K.) followed a selection process, defined prior to the beginning of the review, which included a checklist of inclusion criteria (Table 1). Clinical trial articles about the effects of Kinesio Taping in individuals with an acute ankle sprain were considered eligible for inclusion. The titles and abstracts of all identified articles were assessed to determine whether the study met the inclusion criteria. If this was unclear, the full-text article was retrieved and read to verify its eligibility. Three reviewers (R.V., F.F., and G.M.) determined the final articles to be included in this review. In the event of a disagreement, consensus was reached by discussion.

### 2.3. Data Extraction and Analysis

Data extraction was performed according to the PRISMA guidelines for systematic review, which were modified into the final version for our purposes. The following data were extracted to assess the effect of Kinesio Taping on ankle functional performance: authors, the year of publication, study design, participants’ characteristics (age, gender, and BMI), main follow-up, lesion type, the description of the Kinesio taping protocol, pain outcomes, edema parameters, and the percentage of return to sport. The first reviewer completed the data extraction, whereas the other reviewers checked the accuracy of the extracted data.

### 2.4. Quality Assessment

An eligibility assessment of the articles was performed by two independent reviewers (A.B. and D.G.) to scrutinize for bias. Discrepancies between reviewers were resolved by consensus.

### 2.5. Results Synthesis

A qualitative approach was adopted to present the results of this systematic review. These results are presented in a narrative and illustrative way in the form of figures and tables that condense the relevant information and allow for the easy identification of the main results.

## 3. Results

### 3.1. Search and Literature Selection

**Figure 1:**
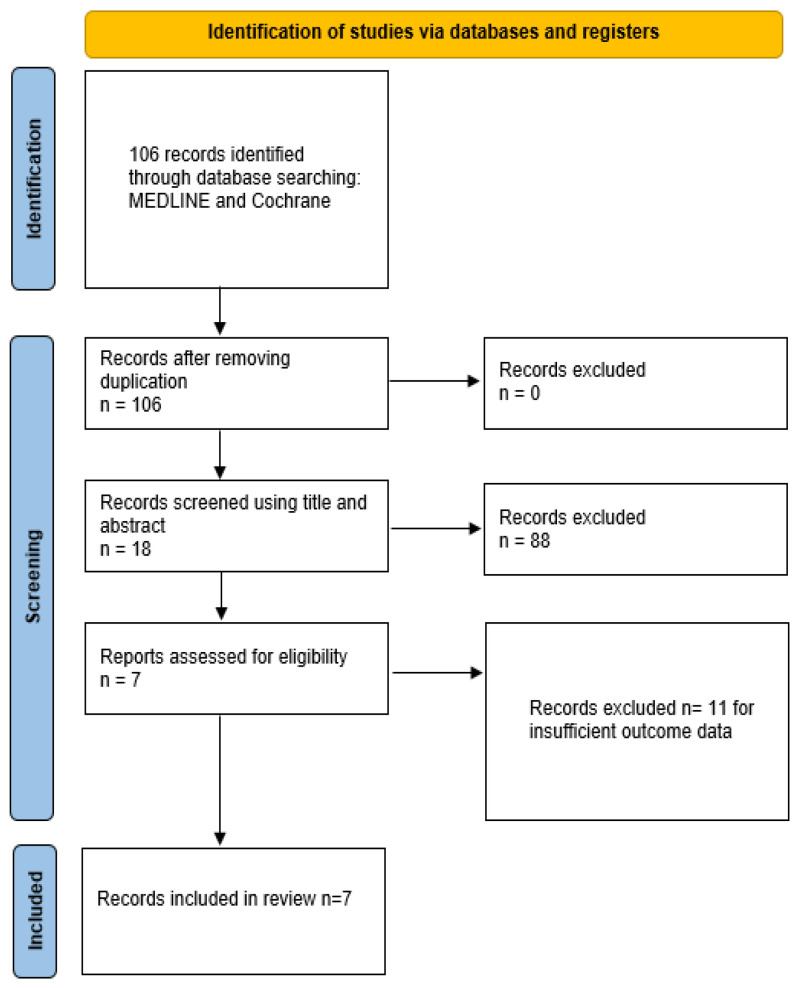
PRISMA flowchart.

The review identified 106 articles relevant to our initial criteria. After applying the exclusion criteria based on study type, methodology, and patient population, 88 articles were excluded. Of the remaining eighteen articles, an additional eleven were excluded due to insufficient outcome data, resulting in the inclusion of seven articles for this review (Figure 1).

### 3.2. Demographics

This systematic review includes data from seven studies, encompassing a total of 247 patients including athletes, physically active individuals, and military personnel who underwent taping treatment for ankle sprain. The mean age of participants varied considerably across the studies, with an average age of 27.98 years; however, one article did not specify the age, although they stated that all enrolled patients were adults. In terms of gender distribution, 164 patients were male (66.39%), and 83 were female (33.61%) (Table 2). Notably, one study did not report the gender distribution.

### 3.3. Follow-Up and Type of Lesion

The review addresses a highly variable follow-up period across the various articles, with an average of 10 days (range of 0–365 days), and three articles did not specify the follow-up duration. Most studies refer to isolated stable grade I and II lesions, although one article does not differentiate between grades, and two articles did not specify the type of lesion. The application of taping was immediate in most studies, with one article specifying an application time of 28.3 days and another at 70.12 h. One article does not mention the time of taping application.

### 3.4. Edema Evaluation

In 2015, Kemler et al. conducted a non-randomized controlled trial to assess the effects of a four-week treatment using soft bracing and taping on 157 adult patients following acute lateral ankle ligament sprains (ALALSs). The study aimed to evaluate the incidence of re-injury and residual symptoms one year after the initial treatment. Within 52 weeks post-trauma, 13 out of 77 participants (17%) in the brace group and 11 out of 80 participants (14%) in the tape group reported a re-injury, resulting in a risk difference of 3.1%. No significant differences were observed between the groups in terms of swelling, functional outcomes, active stability, and pain. The one-year follow-up results indicate that for patients with ALALSs, soft brace and ankle tape treatments yield similar outcomes regarding the recurrence of ALALSs and residual symptoms [15].

Similarly, in 2014, Nunes et al. conducted a randomized controlled trial to evaluate the efficacy of Kinesio Taping in reducing swelling following an acute lateral ankle sprain in 36 athletes. These athletes had sustained an LAS between 48 and 96 h prior to the initial assessment. The participants were randomly assigned to either the experimental group or the control group. The experimental group received a ‘fan cut’ application of Kinesio Taping, while the control group received a 15 cm strip of Kinesio Tape applied in an ‘I’ shape. After three days, there were no significant differences observed between the groups in terms of volumetry, perimetry, or relative volumetry. Nunes et al. concluded that Kinesio Taping is not effective in reducing acute swelling following an LAS in athletes. They recommended further research to study the application of Kinesio Taping for longer durations and at different stages of the inflammatory process [16].

Additionally, Uslu M. et al. conducted a clinical study to determine both the clinical efficacy and antiedema effects of KT and short-leg cast immobilization in acute low-type ankle sprains of physically active patients. Fifty-nine physically active patients were included: thirty-two patients were assigned to the taping group and twenty-seven to the short-leg cast group. Results showed no statistically significant difference between the KT group and the short-leg cast group in terms of ankle edema [17].

### 3.5. Pain Evaluation

Mazloum et al. (2023) compared the effect of ankle taping with neuromuscular electrical stimulation on ankle swelling in athletes with lateral ankle sprains. The results showed no significant difference between the two groups. The study measured the circumference difference between the injured and non-injured ankles on day 0, day 1, and day 15 after the trauma, with results of I-NI (cm) being 0.20 ± 3.90 cm, 0.66 ± 2.83 cm, and 0.63 ± 3.41 cm, respectively [18].

Two of the seven articles focused on evaluating pain before and after the taping treatment using the numerical rating scale (NRS). Karakoyun Ö.F. et al. conducted a quasi-randomized controlled trial to determine the efficacy of KT application as a complementary treatment for patients with an acute ankle sprain (AAS). The study involved 68 adult patients affected by isolated stable grade 1 and grade 2 AASs. Then, they were divided into two groups: a KT group, where Kinesio Tape was applied in addition to the conventional treatment, and a control group, which received only the conventional treatment. Pain intensity, analgesic use, and patient satisfaction were evaluated. Results showed that while pain levels were similar between both groups at baseline and the 30th minute, the control group reported significantly lower pain levels at the 60th minute (*p* = 0.575, *p* = 0.437, and *p* = 0.042, respectively); furthermore, the Kinesio Tape group exhibited reduced drug consumption and higher patient satisfaction levels [19].

Similarly, Shumway J. carried out a case series study on seven active-duty military individuals affected by a high-grade ankle sprain to assess the effect of a combination of manual therapy and rigid sport tape in alleviating pain and increasing patient satisfaction. All patients demonstrated immediate clinically significant decreases in NPRS (numeric pain rating scale) following manual therapy and rigid sport taping, with an average of 4.9 points (mean = 4.9, median = 6, range = 3–8 points). Further analysis shows that the pain and functional improvements were maintained until the next treatment session with an average GROC score (Goblal rating of change) of +3, demonstrating the potential efficacy of this combined treatment approach [20].

### 3.6. Clinical Outcomes

Two of the seven articles conducted a functional assessment using the Karlsson scoring scale, which includes eight categories with a total of 90 points, assessing pain, swelling, instability, stiffness, stair climbing, running, work activities, and support. The study by Witjes et al. (2012), a randomized controlled trial with a one-year follow-up, involved 180 healthy adults with acute, single-sided, first inversion trauma of the lateral ankle ligaments. The participants were divided into three treatment groups: pressure bandage and tape, pressure bandage and brace, and no external support. The group treated with pressure bandage and tape reported a Karlsson scale rating of 60 one year after the trauma [21].

Additionally, Uslu M. et al. found no statistically significant difference in functional scores (AOFAS) between the cohesive taping group and the short-leg casting group during the acute or subacute period following an ankle sprain. However, both treatment methods were effective in improving functional scores [17].

## 4. Discussion

This systematic review evaluates the existing evidence regarding the effectiveness of KT as a treatment for ankle sprains in comparison to other functional treatment methods such as soft bracing, taping, immobilization techniques, and combined treatments involving manual therapy and taping. The studies reviewed presented varying levels of efficacy in addressing this condition, focusing on outcomes like pain relief, functional improvement, and antiedema effects. There was no compelling evidence supporting the effectiveness of Kinesio Taping in improving functional outcomes, such as functional recovery and the return to normal activity levels, especially in the medium and long term. These outcomes were evaluated using two different scales, Karlsson’s scoring scale and the AOFAS, over various follow-up periods. In fact, similar to the findings of Uslu and Witjes, other authors have measured comparable outcomes with similar results. For instance, Lamb et al. [22] assessed ankle function using the foot and ankle outcome score (FAOS) and found clinical benefits at three months for the Aircast brace compared to the elasticated tubular support bandage (Tubigrip) in terms of ankle function quality, but no differences were observed at one and nine months. Five studies utilized Karlsson’s scoring scale (or a modified version of it). Acar et al. [23] assessed ankle function by using the Karlsson scores, pain scores, ankle girth, and additional analgesic needed in 0, 3, 7, and 28 days and found no significant difference between the Kinesio Taping group and the elastic bandage group in all parameters. Boyce et al. [24] employed this scale on day 10 and one month after the ankle sprain, with the patient group treated with an ankle brace scoring higher (indicating better outcomes) than the group treated with an elastic bandage in both measurements. Beynnon et al. [25] used the same scoring scale six months post-injury and found no significant differences between treatment groups; Karlsson et al. [26] also reported no significant differences after 12–24 months of follow-up. Leanderson and Wredmark [27] applied the scale at 3–5 days, as well as at 2, 4, and 10 weeks after the initial injury, similarly finding no significant differences between the group treated with an ankle brace and the group treated with a compression bandage.

The antiedema effect as an outcome variable was investigated in other studies. None of the articles examined, including ours, found statistically significant differences in swelling outcomes. Boyce et al. [24] measured the difference in the edema, in millimeters, between the injured and uninjured ankles on day 10, comparing an elastic bandage group with an Aircast brace group. Twellaar et al. [28] and Neumann et al. [29] assessed the percentage of patients with swelling within the treatment groups; Twellaar et al. did so after an average of 2.3 years of follow-up and Neumann et al. after one year. Dettori et al. [30] measured swelling by volumetry after five weeks. Also, Ruiz-Sanchez et al. [31] stated that using an ankle brace is consistently preferable over Kinesio Tape because the latter fails to offer adequate mechanical support for unstable ankles; therefore, it is preferable to use a cast to treat pain or edema in a grade III ankle sprain for a maximum of 10 days followed by mobilization.

Regarding pain relief, our review found that Kinesio Taping had some positive effects, particularly in reducing the need for pain medication in the short term. Additionally, combining manual therapy with rigid sports tape was effective in alleviating pain. Conversely, different results have been found in other studies. Twellaar et al. [28] evaluated patients 1.8–2.8 years after an ankle injury and found that pain upon palpation was less common in the brace group compared to the tape group (20% vs. 47%). Boyce et al. [24] measured changes in pain scores on a visual analog scale from 0 to 10 between the initial presentation and day 10, finding no significant difference. Finally, Dettori et al. [30] classified pain in different phases, from ‘pain at rest’ (4+) to ‘no pain with running’ (0) and found no differences in effect between bandaging and braces.

However, the lack of standardized parameters across the included studies presents a significant limitation. The heterogeneity in study designs, outcome measures, follow-up durations, and participant characteristics complicates the ability to draw definitive conclusions. Future research must adopt standardized and rigorous methodologies to provide more reliable and conclusive evidence on the effectiveness of Kinesio Taping. Such efforts will be essential in confirming the therapeutic benefits of Kinesio Taping and refining clinical practices for managing acute ankle sprains.

Despite the comprehensive nature of this systematic review on the effects of Kinesio Tape on acute ankle sprain, several significant limitations must be acknowledged. One major issue is the lack of consistent parameters across the included studies, which complicates the ability to draw definitive conclusions. Firstly, the heterogeneity in study designs presents a notable challenge. The studies varied greatly in their sample sizes, control conditions, and intervention protocols. This diversity makes it difficult to directly compare results and determine the overall effectiveness of Kinesio Tape for treating acute ankle sprains. Another limitation is the variation in outcome measures used. Different studies focused on different aspects, such as pain reduction, functional performance, and proprioception. This lack of uniformity makes it challenging to synthesize the findings into a coherent conclusion. The duration of follow-up also varied widely among the studies. Some assessed short-term effects, while others evaluated long-term outcomes. This inconsistency limits our understanding of the sustained efficacy of Kinesio Tape over time.

Participant characteristics were not standardized across the studies. Variations in age, sex, level of physical activity, and severity of the ankle sprain could influence the effectiveness of Kinesio Tape and confound the results. Additionally, not all studies employed adequate blinding of participants and assessors, leading to potential performance and detection bias. This is particularly problematic for subjective outcome measures, such as pain assessment. The presence of proper control groups and standardized placebo conditions was also lacking in some studies. This could affect the reliability of the findings, as the placebo effect is a known confounder in therapeutic interventions. The quality of reporting varied, with some studies lacking detailed descriptions of their methods and results. This inconsistency limits the ability to critically appraise and replicate the studies. Lastly, the exclusive use of articles in English represents another limitation, as it may have led to the exclusion of relevant studies in other languages. Overall, these limitations highlight the need for more rigorous and standardized research methodologies. Future studies should aim to standardize parameters, use consistent outcome measures, and ensure methodological rigor to provide more definitive evidence on the effects of Kinesio Tape on acute ankle sprains.

## 5. Conclusions

Our systematic review found no conclusive evidence supporting the use of Kinesio Taping as a primary treatment. Kinesio Taping alone may not be sufficient to enhance ankle function significantly and reduce edema, especially in the acute phase of an initial treatment combined with the use of short-term anti-inflammatory drugs, and cooling seems to be essential. Clinicians should therefore consider more consistently supported techniques, such as exercise and bracing, which have been shown to improve postural control, movement performance, and neuromuscular control, all of which are critical for ankle function. Kinesio Taping should be viewed as an adjunctive technique rather than a standalone solution, similar to other taping methods.

## Figures and Tables

**Table 1:** Inclusion and exclusion criteria.

	Inclusion	Exclusion
Population	Healthy individuals and patients with ankle sprain	Individuals with non-sports injury: ankle disorder due to nerve, vessel, muscle, etc.
Intervention	Taping applied to ankle joint or gastrocnemius muscle	Non-elastic taping only
Design	Randomized controlled trials, clinic trials and controlled trials	Other designs (e.g., systematic review, opinions commentaries, and case report)
Other	English	Not in English

**Table 2:** Demographic distribution.

Author	Year of Publication	Patients = *n*	Gender W/M	Mean Age y/o
Karakoyun Ö.F. [14]	2024	34	13 W 21 M	29.88 ± 10.23
Mazloum V. [13]	2023	16	0 W 16 M	24.4 ± 1.3
Shumway J.D. [15]	2022	7	1 W 6 M	29.3 ± 7.3
Kemler E. [10]	2015	80	33 W 47 M	31.4
Nunes G.S. [11]	2015	18	5 W 13 M	25
Uslu M. [12]	2015	32	11 W 21 M	27.91 ± 8.31
Witjes S. [16]	2012	60	-	-
Total	-	247	33.61% W 66.39% M	27.90 y/o
